# Altered Heart Rate Regulation in Adolescent Girls and the Vulnerability for Internalizing Disorders

**DOI:** 10.3389/fphys.2018.00852

**Published:** 2018-07-09

**Authors:** Aina Fiol-Veny, Alejandro De La Torre-Luque, Maria Balle, Xavier Bornas

**Affiliations:** ^1^Department of Psychology, University Research Institute on Health Sciences (IUNICS), University of the Balearic Islands, Palma, Spain; ^2^Department of Psychiatry, Mental Health Networking Biomedical Research Centre, Autonomous University of Madrid, Madrid, Spain

**Keywords:** heart rate variability, adolescence, sex, depression, anxiety

## Abstract

**Background:** The association between decreased heart rate variability (HRV) and increased internalizing symptoms is well documented. Adolescence is a critical period for the development of mental health problems, in particular internalizing symptoms. Previous research has illustrated sex differences in adolescent HRV, such that females have reduced short-term resting state HRV compared to males. Studies on long-term ecological recordings of HRV in adolescents are scarce. The aims of the present study were, (a) to test if adolescent females show decreased long-term HRV and cardiac complexity (CC) compared to males, and (b) to explore whether sex and HRV and CC measures, as well as their interaction, would predict internalizing symptoms.

**Materials and Methods:** HRV was recorded in *n* = 166 adolescents (86 girls), on a normal school day. HRV and CC measures were calculated on the interbeat interval time series.

**Results:** Females showed lower HRV and CC in most of the assessed indices. Internalizing symptoms were mainly predicted by HRV whereas sex only predicted symptoms of social anxiety. The interaction between sex and HRV did not predict internalizing symptoms.

**Conclusions:** Results suggest that reduced HRV should be considered as a potential contributor to exacerbating internalizing symptoms in adolescence. Girls with reduced HRV and CC might be prone to the development of internalizing disorders. HRV is a promising tool for the early identification of vulnerability.

## Introduction

Studies have shown that prevalence rates for internalizing disorders (IDs; i.e., depression and anxiety) are higher among females than males (McLean et al., 2011; Beesdo-Baum and Knappe, 2012; Goldman, 2012). Many social and biological factors are discussed to contribute to this discrepancy (Barlow, 2000; Yap et al., 2014). The present study addresses the Negative Valence Systems Domain of the Research Domain Criteria (RDoC) framework (Insel et al., 2010; Insel, 2014) at the physiological autonomic level of analysis, and focuses on sex differences in heart regulation variability and complexity.

Heart rate variability (HRV) refers to the variations in the length of successive interbeat intervals (IBIs), though it is not a unitary concept. By measuring the length of successive intervals, HRV is calculated, and it can be quantified using time domain measures (e.g., the standard deviation of successive intervals or SDNN). However, these measures do not give information about the sources of variability; instead, frequency domain measures of the HRV are used. Thus, use of the high frequency (HF, 0.15–0.40 Hz) band power as an index of vagally mediated HRV is widely accepted, i.e., the variability that depends on the inhibitory action of the autonomic nervous system's parasympathetic branch. The low frequency (LF, 0.4–0.15 Hz) band power has traditionally been interpreted as an index of sympathetic cardiac control. However, recent studies challenge this interpretation and suggest that the LF seems to be a mix of sympathetic, parasympathetic, and other factors (Billman, 2013). Apart from the associations between reduced HF power and IDs (Kemp et al., 2010; Chalmers et al., 2014), a plethora of research has provided support for theoretical models relating reduced vagally mediated HRV and poor emotional regulation (Porges, 1995; Thayer and Lane, 2000, 2009), which is clearly related to many IDs (Aldao et al., 2010). Furthermore, Beauchaine and Thayer (2015) discussed the role of HF HRV as a psychopathological transdiagnostic biomarker within the dimensional framework of the RDoC.

Interest in HRV and cardiac complexity (CC) has grown in the last 30 years. Goldberger (1996) provided a rationale for the need to consider chaos theory and closely related concepts (e.g., fractals) to better understand how physiological systems work, specifically, heart dynamics. Briefly, Goldberger pointed out that classic homeostatic models were unable to explain complex fluctuations in heartbeat. The need to consider the cardiovascular system as a complex system, and specifically the utility of non-linear cardiac measures, has been recently stressed from a clinical perspective by Captur et al. (2017). For a general review on nonlinear, complex biomarkers for emotional disorders (including IDs), see de la Torre-Luque et al. (2016). According to the findings of this meta-analysis, the cardiovascular systems of healthy people show a higher level of CC than the systems of those who suffer from an ID.

The median age of onset for many IDs is during adolescence (Kovacs and Devlin, 1998; Kessler et al., 2005). A recent meta-analysis (Koenig et al., 2017) has shown, through short-term recordings, that adolescent females display lower vagal activity and a higher mean heart rate (HR) than adolescent males under resting conditions. Several studies have also correlated reduced HRV with internalizing symptoms. Dietrich et al. (2007) found that higher HR and lower respiratory sinus arrhythmia (the influence of respiration on HR and another index of the vagally mediated HRV) were related to increased internalizing symptoms, even in healthy adolescents, and Greaves-Lord et al. (2010) found that low respiratory sinus arrhythmia predicted anxiety levels in healthy adolescent females 2 years later, but not in males. Few studies have looked at sex differences using longer cardiac recordings. Faulkner et al. (2003) studied sex differences in HRV measurements taken from 24-h ECG recordings in early adolescents (*n* = 43, mean age = 15), and found lower SDNN values for females, but no differences in HF power. Silvetti et al. (2001) used several time domain measures of HRV and found higher SDNN in males in 24-h recordings from 103 children and adolescents (1–20 years old), though no sex differences were found in other measures (e.g., RMSSD, root mean squares of successive IBI differences). Bobkowski et al. (2017) did not find sex differences in HRV or in CC measures derived from Poincaré plots calculated from 24-h recordings from 100 children (aged 3-18 years old). Our previous study (Fiol-Veny et al., 2018) examined sex- and anxiety-related differences in HRV and CC on adolescents with high- versus low- anxiety scores (n = 95, mean age = 14) through long cardiac ecological recordings. There was no interaction between sex and anxiety, but adolescents with high anxiety symptoms showed lower HRV than their low-anxious counterparts, whereas females showed lower HRV and lower CC than males. However, we underlined that future research should also investigate sex-related differences regardless of the level of anxiety, i.e., assessing boys and girls that not only encompass high and low-anxiety students, but all ranges of anxiety symptom levels.

The wide age range of the samples in some of these studies and the methodological differences (e.g., length and conditions of the ECG recordings or the specific measure of HR regulation) could explain the incongruence of results, although most of them point out that cardiac regulation is less flexible in adolescent girls compared to boys. Importantly, HRV and CC in regular school settings (instead of more conventional lab settings) have rarely been studied, though this ecological assessment could provide helpful knowledge about heart regulation in everyday conditions (see Bornas et al., 2015).

Therefore, the present study aimed to: (a) to test if adolescent females show decreased HRV and CC compared to males and (b) to explore whether sex and measures of HRV and CC, as well as their interaction, predict levels of internalizing symptoms.

## Materials and methods

The Bioethics Committee at the university approved of all procedures, and all participants and their parents or legal guardians provided written consent.

### Participant selection

Data for the present analyses were taken from a sizable longitudinal project (TRANS; Bornas et al., 2014), the general aim of which was to examine the trajectories of anxiety symptoms and physiological correlates of anxiety in adolescents over the course of 3 years. Self-reported symptoms of anxiety were assessed by repeated measurements every 6 months. All participants were middle-class Caucasians from both urban and rural areas on the island of Majorca, Spain. Unlike our previous study from the TRANS project (Fiol-Veny et al., 2018), participants' selection encompassed not only those with extremely high or low scores of anxiety symptoms. The full range of scores was covered in the present study to overcome this limitation, and a much larger and non-overlapping sample was used. According to Quintana (2017), a sample size of *n* = 82 per group is required to achieve a statistical power of 90% to detect effect sizes in HRV case-control studies. In order to achieve this sample size, and considering the average number of students per school enrolled in the TRANS project, five of the 12 schools were randomly selected and invited to participate in the present study. All of them agreed, and therefore data from 169 adolescents were collected. The exclusion criteria were: suffering from severe mental retardation; a neurological, developmental, or psychiatric disorder (American Psychiatric Association, 2000); or any severe cardiovascular or respiratory disease, as per medical records that were reported by parents. As we examined HRV and CC in a sample of healthy adolescents, students with any ID diagnoses were excluded (*n* = 3). The final sample (*N* = 166) comprised *n* = 86 girls and *n* = 80 boys.

### Procedure

First, we administered an online version of the self-report questionnaire (see instruments below) to all participants. In the following month we conducted cardiac recordings on a regular school day within the academic context but only when participants were not going to be doing any physical or out-of-the-ordinary activities during the time of the recording. Participants left the classroom in the early morning (around 8:00 a.m.) and went to a private room to have their BMIs calculated via measurements of height and weight. Subsequently, the researcher put two electrodes on each participant's chest and attached a portable device to the electrodes (see the Cardiac Assessment section). Participants then returned to their classrooms with the devices activated, and cardiovascular function was continuously measured for the first 120 min of that day. Finally, each participant was scheduled for a structured interview with one of the researchers. Adolescents who had consumed alcohol, drugs, and/or caffeinated beverages during the previous 4 h, adolescents with an acute illness, or those who were menstruating were assessed another day.

### Instruments

#### Psychological assessment

We used the Revised Child Anxiety and Depression Scale (RCADS; Chorpita et al., 2000) to assess anxiety and depression symptoms. It is a 47-item, self-report questionnaire, with sub-scales assessing separation anxiety disorder, social anxiety, generalized anxiety disorder, panic disorder, obsessive compulsive disorder, and major depressive disorder. There is an overall scale indicating the total level of anxiety symptomatology. The RCADS requires respondents to rate how often each item applies to them. Items are scored 0–3, with 0, 1, 2, and 3, respectively corresponding to “never,” “sometimes,” “often,” and “always.” The internal consistency of the subscales within the present sample ranged from α = 0.80 to α = 0.94.

The Mini-International Neuropsychiatric Interview for Children and Adolescents (M.I.N.I. Kid; Sheehan et al., 1998) was used to determine if an ID was present. This is a structured diagnostic interview for children from 6 to 17 years of age based on the Diagnostic and Statistical Manual of Mental Disorders, Fourth Edition, Text Revision (American Psychiatric Association, 2000) and the International Statistical Classification of Diseases, 10th Revision (World Health Organization, 1992).

Participants filled out other questionnaires required by the TRANS study: The Early Adolescence Temperament Questionnaire (EATQ-Revised long form; Ellis and Rothbart, 2001) and the Sensitivity to Punishment and Sensitivity to Reward Questionnaire Junior version (SPSRQ-J; Torrubia et al., 2008).

#### Cardiac assessment

The reporting of cardiac measures follows GRAPH guidelines (Quintana et al., 2016). The Firstbeat Bodyguard 2© (Firstbeat Technologies Ltd., Jyväskylä, Finland) is a measurement device that is attached to the skin with two electrodes: one on the left side of the chest and the other on the right side under the collarbone. The device continuously records beat-to-beat HR (i.e., normal to normal intervals or IBIs) with a sampling frequency of 1,000 Hz; the recordings lasted 120 min. The first and last 15 min of each time series were removed to eliminate the adaptation period and the period when participants stopped their regular activity. Thus, each time series analyzed was 90 min long, and the number of IBIs in each time series depended on the participant's HR. We visually inspected each recording to exclude distorted signals due to apparatus failure (*n* = 2 out of *n* = 168, 1.19%). The time series was filtered with the Physionet (Goldberger et al., 2000) HRV toolkit (http://www.physionet.org/tutorials/hrv-toolkit/) a low-pass band filter at 1,100 ms, a high-pass band filter at 400 ms, and a central interval filter were applied. Using a window of 11 intervals (5 intervals on either side of the central interval) but excluding the central interval, the average over the window was calculated. If the central interval laid outside 20% (0.20) of the window average, this interval was excluded and the window was advanced to the next interval, resulting in the mean of excluded IBIs (*n* = 229 out of *n* = 11808, 1.94%).

##### HRV calculation

Time domain measures were based on the beat-to-beat (or normal to normal, NN) intervals. We used the AVNN (average of all NN intervals), a measure of HR, and RMSSD.

Frequency domain measures provided the total spectral power of all NN intervals in each frequency band by transforming the time series into the frequency domain through the fast Fourier transform. LF ranges from 0.04 to 0.15 Hz and HF from 0.15 to 0.40 Hz.

All HRV measures were calculated using the Physionet (Goldberger et al., 2000) HRV toolkit.

##### CC calculation

The detrended fluctuation analysis (DFA) is a method designed by Peng et al. (1995), which allows for the estimate of temporal power-law form correlations embedded in IBI time series. We obtained two scaling exponents: the α1 exponent, which reflects short-term scaling (4-11 beats), and the α2 exponent, which reflects long-term scaling (>11 beats). α1 > α2 in healthy individuals. For uncorrelated data α = 0.5, while 0.5 < α < 1 indicates persistent long-range power-law correlations. Both exponents were calculated using Kubios HRV Software, version 2.1 (Tarvainen et al., 2014).

For the fractal dimension (FD), the allometric aggregation method examines the invariance of the relationship between the mean and the standard deviation of a data series as the data points are iteratively aggregated, thus decreasing the resolution (scale) of the series. First, the mean and standard deviation of the time series of length *N* (*x*_1_, *x*_2_, *x*_3_,…, *x*_n_) are calculated. Secondly, each pair of adjacent points is aggregated (*x*_1_ + *x*_2_, *x*_3_ + *x*_4_,…, *x*_n−1_ + *x*_n_) to get a time series of length *N*/2, and the mean and standard deviation of that time series are calculated. We repeate this aggregation process for 3, 4, 5, and usually up to *N*/10 adjacent data points. Since our IBI time series included around 9000 values, we repeated the aggregation process for 1, 10, 20, 30, 40, 50, 60, 70, 80, 90, and 100 adjacent data points and then plotted the mean and standard deviation of each level of aggregation on a logarithmic graph. When the number of aggregated values increases, the relationship between mean and standard deviation remains the same (invariant) and can therefore be described as a linear relationship, with the slope of the line being the scaling exponent *h*. Notice that the scaling exponent for random fluctuations is 0.50 and for deterministically regular processes it is 1 (in both cases the time series would not be self-similar or fractal). If we take the variance instead of the standard deviation, the slope of the line is 2*h*. A MathWorks Matlab code (available upon request) was developed to calculate the exponent. The fractal dimension is then calculated according to the formula FD = 2-*h*.

The multiscale entropy analysis (Costa et al., 2002) evaluates the entropy (or irregularity) of complex time series at different time scales. It creates consecutive coarse-grained time series by averaging a successively increasing number of data points in non-overlapping windows. Then, sample entropy (SampEn; Richman and Moorman, 2000), which is the negative natural logarithm of the conditional probability that two sequences with similar *m* points remain similar at the next point, is calculated for each of the coarse-grained time series. Less entropy implies higher regularity and predictability. In the current study, entropy for scale factors 1, 5, 10, 15, and 20 was calculated using the software available from https://physionet.org/physiotools/mse/.

### Analytical strategy

According to recommendations made by Quintana (2017) regarding the sample size required to detect medium effect sizes in HRV studies with a statistical power > 80%, around 80 participants of both sexes should be allocated to each group. We explored differences in age and BMI between males and females, through between-group *t*-tests, and differences in internalizing symptoms using one-way MANOVAs. The alpha level was adjusted for multiple comparisons (Bonferroni) and set to 0.007. Since HF power values were not normally distributed (Shapiro-Wilk's *W* = 0.885, *p* < 0.001, skewness = 1.49, kurtosis = 3.10), we took the natural logarithm of these values (lnHF) before conducting statistical analyses that require data to be normally distributed. After this transformation, the lnHF distribution was normal (*W* = 0.98, *p* > 0.05, skewness = −0.47, and kurtosis = 0.31). Cardiac measures were adjusted by the average HR to control for HR-derived mathematical bias. The cardiac measures that correlated negatively with HR were divided by the average HR. Those which correlated positively (only DFA 1) were multiplied by the average HR, in line with Gasior et al. (2016) and Sacha et al., 2013a,b. All subsequent statistical analyses were conducted with both adjusted and unadjusted cardiac measures.

We analyzed differences in cardiac measures between males and females using one-way MANOVAs. Then, Cohen's *d* effect size values were calculated, and they were interpreted according to Quintana (2017). The research team grouped the cardiac measures into four sets of MANOVAs, and alpha levels were corrected according to Bonferroni: with time domain measures in the first set (*p* < 0.025), frequency-domain measures in the second set (*p* < 0.025), DFA and FD in the third set (*p* < 0.017), and entropy measures in the fourth set (*p* < 0.01). Multiple linear regressions analyses were also performed with each internalizing symptoms subscale as the dependent variable, and both sex and cardiac measures as predictors. In step 1, sex (coded 1 for boys, and 2 for girls) and each cardiac measure were entered as predictors. In step 2, the interaction between sex and each cardiac measure was added. To prevent for multicollinearity, values of cardiac measures were standardized. All analyses were conducted using the IBM SPSS Statistics v.20.0.0 package.

## Results

The sample in the present study was comprised of two groups: 86 girls (mean age *M* = 14.96, *SD* = 0.46, range = 13.78–16.40 years old; mean body mass index [BMI] = 17.68, *SD* = 2.74) and 80 boys (mean age *M* = 14.96, *SD* = 0.36, range = 14.42–16.26 years old; mean BMI = 18.19, *SD* = 3.35).

No significant differences in age or BMI were found between males and females. Sex differences in cardiac measures are shown in Table 1. Females showed higher mean HR and decreased variability in both time-domain and frequency-domain measures when compared to males (*p* < 0.025, according to the Bonferroni correction). Females also showed lower CC than males (*p* < 0.017 for the FD, and *p* < 0.010 for sample entropy measured at scale factors 15 and 20). The effect sizes can be considered medium as most of them were close to 0.50. When we analyzed adjusted cardiac indices, similar results were obtained but the differences between males and females increased. Furthermore, differences also became significant for the α2 exponent (*p* = 0.002, *d* = 0.49) and entropy at scale factors 5 (*p* = 0.006, *d* = 0.44) and 10 (*p* = 0.002, *d* = 0.50).

**Table 1:** Mean, standard deviation (in parenthesis) and comparison of cardiac measures (unadjusted by HR) between males and females.

	**Males (*n* = 80)**	**Females (*n* = 86)**	***F*_(1, 164)_**	***p***	**Cohen's *d***
HR	89.33 (11.006)	93.421 (10.407)	6.11	0.014	0.39
RMSSD	38.16 (13.89)	33.24 (12.31)	5.93	0.016	0.38
LF	1913.67 (767.09)	1614.65 (686.96)	7.10	0.008	0.42
lnHF	6.26 (0.69)	5.96 (0.76)	6.71	0.010	0.41
DFAα1	1.40 (0.14)	1.42 (0.13)	1.37	0.243	0.18
DFAα2	0.98 (0.08)	0.96 (0.07)	4.30	0.040	0.32
FD	1.10 (0.03)	1.08 (0.02)	11.60	0.001	0.53
MSE sf1	0.90 (0.23)	0.87 (0.19)	0.87	0.351	0.15
MSE sf5	1.51 (0.19)	1.47 (0.17)	2.96	0.087	0.27
MSE sf10	1.53 (0.13)	1.49 (0.13)	3.63	0.058	0.30
MSE sf15	1.51 (0.12)	1.45 (0.12)	9.06	0.003	0.47
MSE sf20	1.49 (0.12)	1.43 (0.12)	10.14	0.002	0.50

*HR, Heart rate (beats per minute); RMSSD, square root of the mean of the squares of differences between adjacent NN intervals (ms); LF, low frequency power (ms^2^); lnHF, logarithmic transformation of high frequency power; DFA α1, alpha 1 exponent calculated by Detrended Fluctuation Analyses; DFA α2, alpha 2 exponent calculated by Detrended Fluctuation Analyses; FD, Fractal Dimension; MSE sf1, multiscale entropy scale factor 1; MSE sf5, multiscale entropy scale factor 5; MSE sf10, multiscale entropy scale factor 10; MSE sf15, multiscale entropy scale factor 15; MSE sf20, multiscale entropy scale factor 20*.

Means, standard deviations, and ranges of total internalizing symptoms are shown in Table 2 and separated by sex. Sex differences in internalizing symptoms are also presented in Table 2. No significant differences were found between males' and females' internalizing symptoms (*p* > 0.007).

**Table 2:** Mean, standard deviation and range of total internalizing symptoms, and separated between males and females.

	**Total**			**Males (*****n*** = **71)**			**Females (*****n*** = **83)**					
**Variable**	***M***	***SD***	**Range**	***M***	***SD***	**Range**	***M***	***SD***	**Range**	***F* (1, 152)**	***p***	**Cohen's *d***
Social anxiety	8.85	5.53	0–25	7.62	4.86	0–25	9.92	5.88	0–25	6.83	0.010	0.43
Generalized anxiety	5.82	3.61	0–15	5.69	3.61	0–15	5.94	3.63	0–15	0.18	0.670	0.07
Separation anxiety	1.56	2.49	0–16	1.62	2.79	0–16	1.51	2.22	0–10	0.08	0.928	0.05
Depression	8.04	5.64	0–23	7.25	5.23	0–23	8.71	5.92	0–23	2.58	0.110	0.26
Obsessive compulsive	3.11	3.35	0–16	3.07	3.25	0–16	3.14	3.45	0–15	0.02	0.892	0.02
Panic	4.71	4.85	0–21	4.67	4.94	0–21	4.75	4.79	0–19	0.01	0.928	0.02
Total anxiety	24.06	16.21	0–83	22.67	15.79	0–83	25.25	16.55	0–76	0.97	0.327	0.16

Hierachical multiple regression analyses predicting internalizing symptoms from sex and unadjusted cardiac measures are shown in Table 3 (HRV measures) and Table 4 (CC measures). For unadjusted cardiac data, sex was a significant predictor, but of social anxiety only. HR predicted social anxiety, panic, obsessive-compulsive, depression, and total anxiety symptoms. LnHF predicted social anxiety, depression and total anxiety symptoms. Depression was also predicted by the α1 exponent entropy at scale factors 1 and 5. We also found interaction effects between sex and the α1 exponent in the prediction of depression symptoms. The interaction between sex and entropy at scale factors 5 and 10 also predicted separation anxiety symptoms.

**Table 3:** Hierachical multiple regression analyses predicting internalizing symptoms from sex and HRV measures (unadjusted by HR).

	**Internalizing symptomatology**													
	**Social A**		**GAD**		**Separation A**		**PD**		**OCD**		**Depression**		**Total anxiety**	
**Predictor**	**Δ*R*^2^**	**β**	**Δ*R*^2^**	**β**	**Δ*R*^2^**	**β**	**Δ*R*^2^**	**β**	**Δ*R*^2^**	**β**	**Δ*R*^2^**	**β**	**Δ*R*^2^**	**β**
Step 1	0.068^**^		0.004		0.025		0.040^*^		0.081^**^		0.055^*^		0.050^*^	
Sex		0.183^*^		0.026		−0.046		−0.023		−0.032		0.099		0.048
HR		0.161^*^		0.056		0.157		0.202^*^		0.287^**^		0.199^*^		0.212^**^
Step 2	0.001		0.000		0.001		0.002		0.000		0.001		0.000	
Sex × HR		0.092		0.068		−0.090		−0.127		0.006		−0.097		−0.004
Step 1	0.066^**^		0.007		0.008		0.016		0.020		0.037		0.028	
Sex		0.184^*^		0.023		−0.036		−0.012		−0.011		0.107		0.057
RMSSD		−0.155		−0.076		−0.085		−0.127		−0.144		−0.144		−0.151
Step 2	0.016		0.020		0.002		0.004		0.015		0.016		0.016	
Sex × RMSSD		−0.394		−0.437		−0.136		−0.203		−0.388		−0.392		−0.394
Step 1	0.061^**^		0.005		0.002		0.001		0.006		0.021		0.014	
Sex		0.186^*^		0.025		−0.028		0.004		−0.002		0.118		0.066
LF		−0.136		−0.059		−0.032		−0.023		−0.081		−0.068		−0.088
Step 2	0.003		0.010		0.000		0.000		0.004		0.000		0.003	
Sex × LF		−0.163		−0.316		0.048		−0.037		−0.197		−0.020		−0.170
Step 1	0.069^**^		0.011		0.009		0.021		0.024		0.046^*^		0.034	
Sex		0.182^*^		0.020		−0.037		−0.015		−0.013		0.103		0.054
lnHF		−0.165^*^		−0.098		−0.093		−0.147		−0.156		−0.173^*^		−0.168^*^
Step 2	0.010		0.008		0.000		0.004		0.012		0.010		0.009	
Sex × lnHF		−0.331		−0.302		−0.006		−0.205		−0.364		−0.333		−0.318

*N = 154. HR, heart rate (beats per minute); RMSSD, square root of the mean of the squares of differences between adjacent NN intervals; LF, low frequency power; lnHF, logarithmic transformation of high frequency power; Social A, social anxiety symptoms; GAD, generalized anxiety symptoms; Separation A, separation anxiety symptoms; PD, panic symptoms; OCD, obsessive compulsive symptoms*.

* *p < 0.05*;

** *p < 0.01*.

**Table 4:** Hierachical multiple regression analyses predicting internalizing symptoms from sex and CC measures (unadjusted by HR).

	**Internalizing symptomatology**													
	**Social A**		**GAD**		**Separation A**		**PD**		**OCD**		**Depression**		**Total anxiety**	
**Predictor**	**Δ*R*^2^**	**β**	**Δ*R*^2^**	**β**	**Δ*R*^2^**	**β**	**Δ*R*^2^**	**β**	**Δ*R*^2^**	**β**	**Δ*R*^2^**	**β**	**Δ*R*^2^**	**β**
Step 1	0.064^**^		0.007		0.007		0.037		0.020		0.049^*^		0.034	
Sex		0.196^*^		0.028		−0.029		−0.008		0.000		0.115		
DFAα1		0.144		0.079		0.081		0.192^*^		0.140		0.180^*^		0.166^*^
Step 2	0.016		0.015		0.002		0.007		0.014		0.034^*^		0.016	
Sex × DFAα1		0.393		0.387		0.144		0.259		0.379		0.579^*^		0.398
Step 1	0.045^*^		0.003		0.001		0.000		0.002		0.022		0.007	
Sex		0.200^*^		0.041		−0.019		0.006		0.004		0.141		0.077
DFAα2		−0.050		0.043		0.023		−0.006		−0.049		0.077		−0.016
Step 2	0.004		0.000		0.017		0.003		0.001		0.002	−0.132		
Sex × DFAα2		−0.196		−0.069		−0.411		−0.162		−0.120		0.005		−0.219
Step 1	0.073^**^		0.007		0.004		0.003		0.007		0.030		0.020	
Sex		0.188^*^		0.031		−0.037		−0.005		−0.001		0.123		0.064
FD		−0.154		−0.069		−0.056		−0.056		−0.082		−0.097		−0.110
Step 2	0.001		0.003		0.019		0.001		0.000		0.001		0.000	
Sex × FD		−0.081		−0.169		0.437		−0.104		0.066		−0.102		−0.015
Step 1	0.055^*^		0.002		0.019		0.030		0.033		0.043^*^		0.030	
Sex		0.203^*^		0.033		−0.029		0.000		0.003		0.122		0.073
MSEsf1		−0.110		−0.025		−0.136		−0.172^*^		−0.182^*^		−0.163^*^		−0.153^*^
Step 2	0.000		0.000		0.010		0.002		0.002		0.000		0.001	
Sex × MSEsf1		−0.030		−0.048		0.316		0.121		0.135		−0.054		0.113
Step 1	0.056^*^		0.012		0.018		0.015		0.021		0.048^*^		0.028	
Sex		0.196^*^		0.024		−0.036		−0.005		−0.004		0.111		0.065
MSE sf5		−0.113		−0.104		−0.135		−0.124		−0.145		−0.177^*^		−0.150
Step 2	0.001		0.003			0.553^*^		0.242		0.314		0.139	0.009	
Sex × MSEsf5		0.107		0.169		0.031^*^		0.006		0.010		0.002		0.296
Step 1	0.049^*^		0.008		0.007		0.000		0.002		0.019		0.011	
Sex		0.197^*^		0.023		−0.034		0.008		0.006		0.123		0.071
MSE sf10		−0.081		−0.086		−0.084		0.003		−0.039		−0.047		−0.067
Step 2	0.001		0.002		0.034^*^		0.010		0.010		0.003		0.010	
Sex × MSEsf10		−0.091		−0.155		0.608^*^		0.330		0.323		0.192		0.325
Step 1	0.051^*^		0.010		0.006		0.000		0.001		0.018		0.010	
Sex		0.187^*^		0.014		−0.039		0.012		0.003		0.121		0.065
MSE sf15		−0.094		−0.094		−0.072		0.022		−0.036		−0.037		−0.065
Step 2	0.000		0.001		0.018		0.002		0.006		0.000		0.003	
Sex × MSEsf15		−0.026		0.106		0.458		0.147		0.260		0.062		0.201
Step 1	0.049^*^		0.010		0.006		0.000		0.004		0.020		0.011	
Sex		0.189^*^		0.012		−0.041		0.010		−0.005		0.115		0.063
MSE sf20		−0.077		−0.096		−0.078		0.014		−0.067		−0.063		−0.069
Step 2	0.000		0.000		0.002		0.001		0.003		0.000		0.000	
Sex × MSEsf20		−0.055		0.043		0.163		−0.098		0.182		0.007		0.024

*N = 154. DFA α1, alpha 1 exponent calculated by Detrended Fluctuation Analyses; DFA α2, alpha 2 exponent calculated by Detrended Fluctuation Analyses; FD, Fractal Dimension; MSE sf1, multiscale entropy scale factor 1; MSE sf5, multiscale entropy scale factor 5; MSE sf10, multiscale entropy scale factor 10; MSE sf15, multiscale entropy scale factor 15; MSE sf20, multiscale entropy scale factor 20; Social A, social anxiety symptoms; GAD, generalized anxiety symptoms; Separation A, separation anxiety symptoms; PD, panic symptoms; OCD, obsessive compulsive symptoms*.

* *p < 0.05*;

** *p < 0.01*.

**Figure 1:**
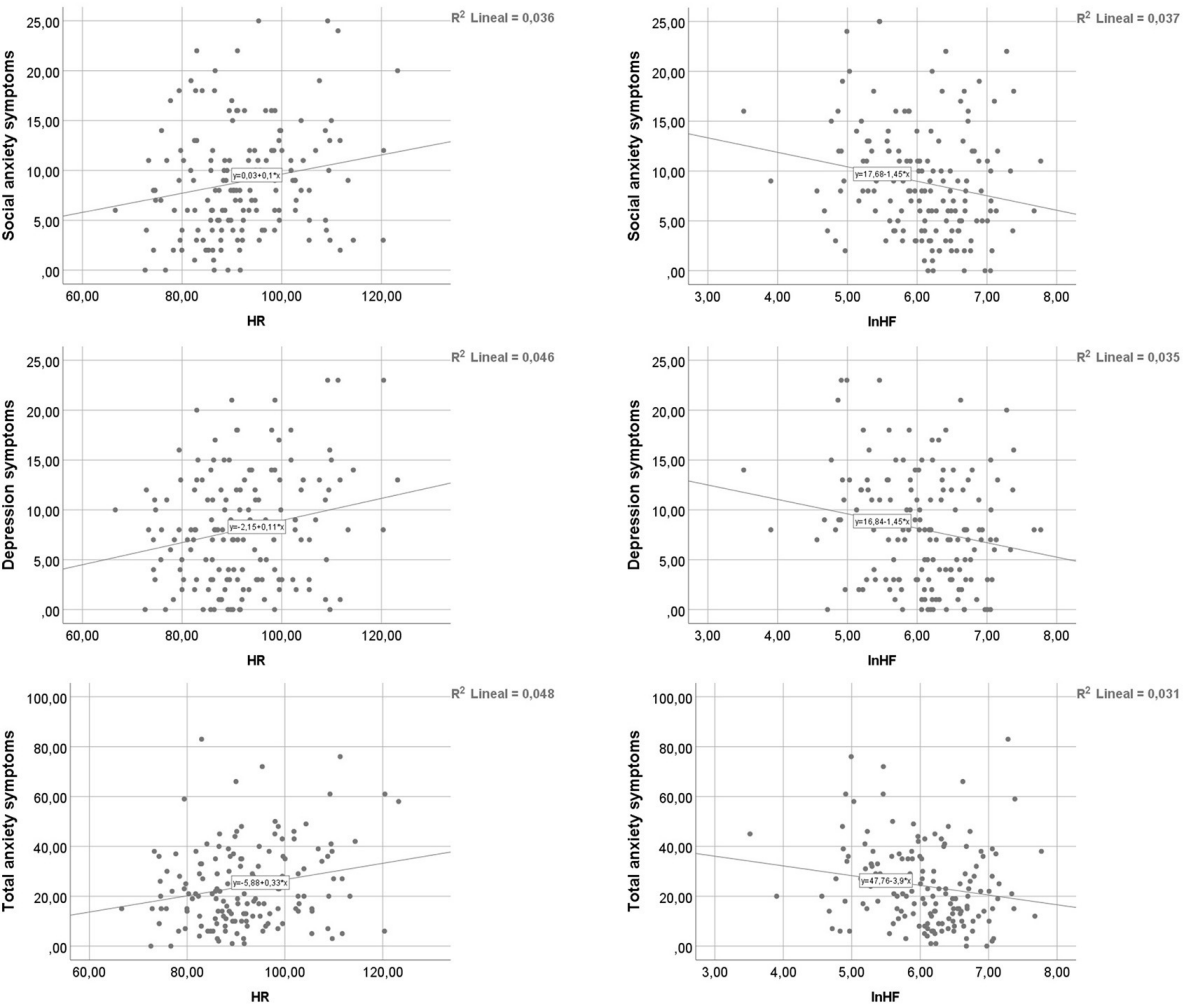
Scatter plots of HR and lnHF with social anxiety **(upper panels)**, depressive **(medium panels)**, and total anxiety **(lower panels)**.

Scatter plots of HR and lnHF (both unadjusted by HR) with social anxiety, depressive and total anxiety symptoms are shown in Figure 1.

When we adjusted cardiac indices by HR, sex was a significant predictor of social anxiety symptoms (β ranged from 0.163 to 0.195, *p* < 0.05). HR predicted social anxiety (β = 0.161, *p* = 0.044), panic (β = 0.202, *p* = 0.013), obsessive-compulsive symptoms (β = 0.287, *p* = 0.000), depression (β = 0.199, *p* = 0.014), and total anxiety (β = 0.212, *p* = 0.009). LnHF predicted social anxiety (β = −0.169, *p* = 0.036), panic (β = −0.183, *p* = 0.026), obsessive-compulsive symptoms (β = −0.217, *p* = 0.008), depression (β = −0.182, *p* = 0.026), and total anxiety (β = −0.190, *p* = 0.020). The α1 exponent was a significant predictor for social anxiety (β = 0.186, *p* = 0.020), panic (β = 0.235, *p* = 0.004), obsessive-compulsive symptoms (β = 0.269, *p* = 0.001), depression (β = 0.233, *p* = 0.004), and total anxiety (β = 0.232, *p* = 0.004). The α2 exponent predicted panic (β = −0.166, *p* = 0.045), obsessive-compulsive symptoms (β = −0.238, *p* = 0.004), and total anxiety (β = −0.171, *p* = 0.038). The FD was a significant predictor for panic (β = −0.177, *p* = 0.031), obsessive-compulsive symptoms (β = −0.246, *p* = 0.002), depression (β = −0.173, *p* = 0.033), and total anxiety (β = −0.184, *p* = 0.024). Entropy at all scale factors predicted obsessive-compulsive (β from −0.241 to −0.204, *p* < 0.05) and total anxiety (β from −0.202 to −0.163, *p* < 0.05) symptoms. Social anxiety symptoms were predicted by entropy at scale factors from 5 to 20 (β from −0.178 to −0.160, *p* < 0.05). Separation anxiety symptoms were predicted by entropy at scale factor 5 (β = −0.168, *p* = 0.041). Panic symptoms were predicted by entropy at scale factors 1 and 5 (β = −0.177, *p* = 0.029 and β = −0.185, *p* = 0.024, respectively). Finally, depression symptoms were predicted by entropy at scale factors 1, 5, and 20 (β = −0.158, *p* = 0.050; β = −0.202, *p* = 0.013; and β = −0.175, *p* = 0.035, respectively). No interaction effects of sex and cardiac measures were found.

## Discussion

Reduced HRV is associated with IDs (Kemp et al., 2010; Chalmers et al., 2014). The present study aimed to examine sex differences in HR regulation in middle adolescence, a typical time of ID onset (Kovacs and Devlin, 1998; Kessler et al., 2005). We followed a flexible, non-categorical approach to study the ID spectrum (following RDoC framework and under the Negative Valence Systems Domain), considering the physiological unit of analysis. Since the development of these disorders takes places in our daily lives, we decided to record cardiac activity during participants' routine school activities.

Sex differences were found in most of the HRV measures that support the main hypotheses. Females showed higher and less variable HR in the time domain, i.e., the length of successive IBIs did not change as much as in males. When we looked at the spectral components of HR, we found decreased vagal activity (lower HF power) in females. Low vagally-mediated HRV has been repeatedly reported to be associated with anxiety (see Chalmers et al., 2014; Bornas et al., 2015) and depression (Kemp et al., 2010). In our study, females showed lower LF power. The meaning of the differences in LF is far from clear (Billman, 2013), but our results show that the vagal influence on the HR in everyday life at school is lower in adolescent females compared to males.

Previous studies using 24-h ECG recordings have provided inconclusive results. Bobkowski et al. (2017) did not find sex differences in HRV or in CC, whereas Faulkner et al. (2003) and Silvetti et al. (2001) reported higher SDNN in males than in females. However, we must note that the ages of the samples in these studies are hardly comparable: Faulkner et al. (2003) studied 75 adolescents, aged 15 years old (*SD* = 1.6 years); Silvetti et al. (2001) included 103 participants from 1 to 20 years old; and Bobkowski et al. (2017) studied 100 children and adolescents aged 3-18. The age group in this latter study cannot be properly compared to Faulkner's study sample. The age range of the current study sample was much closer to Faulkner's, and the results confirm the same findings using a much larger sample size, i.e., females show reduced HRV when compared to males. The meta-analysis conducted by Koenig et al. (2017) is similar in that females displayed lower vagally-mediated HRV in short recordings under resting conditions. However, a direct comparison of these studies and the current study should be made with caution due to the different conditions in which the cardiac recordings were taken. A more proper comparison could be done with our previous study (Fiol-Veny et al., 2018), which was also based on 90 min long ecological recordings. It was found that adolescent females (mean age = 14) showed lower HRV and CC than males. However, we took an a priori groups-based approach, selecting adolescents above the 75th percentile or below the 25th percentile in anxiety symptomatology. The current study overcomes this limitation, considering all ranges of anxiety symptomatology (and also depression symptoms), and using a non-overlapping and larger sample size. Importantly, the previously reported differences between males and females are confirmed by the current results.

Considering the complex regulation of HR, we found the DFA α2 exponent to be higher (closer to 1) in males than in females, but only when adjusted by HR. DFA quantifies the fractal-like properties of IBI time series data. The α2 exponent gives a measure of these properties for long-range (>11 beats) correlations, and Peng et al. (1995) showed that α2 = 1 in healthy people. Though statistically significant, the difference between females and males could be physiologically minimal as both exponents (adjusted by HR) were close to 0.010 (0.011 for males and 0.010 for females, Cohen's *d* = 0.49).

We should look at the results obtained through allometric aggregation to complete this picture. These results revealed significant sex differences (Cohen's *d* = 0.46 for adjusted and Cohen's *d* = 0.53 for unadjusted data) in that the FD of males' HR was higher than the FD of females (and therefore, heart regulation seemed to be more complex in males than in females). Again, the physiological meaning of the small differences in the absolute mean values prevents a clear interpretation of this finding, but both the α2 exponents and the FD values point to poorer HR regulation in females. Furthermore, sex differences in HR entropy were clear at scale factors 15 and 20 with unadjusted data, and at scale factors from 5 to 20 with adjusted data, with females showing more regular and predictable HRs. By increasing the scale factor, the differences also increased (Cohen's *d* = 0.15 for scale factor 1, and *d* = 0.50 for scale factor 20). Together, these findings clearly underline the more complex regulation of HR in males than in females. We should say that HR scaling properties underlie the role of vagal activity (indexed in this study by the HF band power), as pointed out by Balle et al. (2015). Hence, it is not only the real-time function of the parasympathetic system that is reduced in females, but the HR fractal properties, too.

The second aim of this study was to explore whether sex and measures of HRV and CC, as well as their interaction, predicted levels of internalizing symptoms. We should say that no prediction analysis could be performed in our previous study (Fiol-Veny et al., 2018) because of its methodological design. Therefore, the current study adds important information on the predictive role of HRV and CC on several internalizing symptoms. For unadjusted data, the regression models showed that social anxiety symptoms were predicted by HR and lnHF. Depression symptoms were predicted by HR, lnHF, the α1 exponent, and entropy at scale factors 1 and 5. Panic, obsessive-compulsive, and total anxiety symptoms were also predicted by HR. The regression models for adjusted cardiac indices showed that panic, obsessive-compulsive symptoms, depression, and total anxiety were predicted by HR, lnHF, the α1 exponent, the FD, and entropy at scale factors 1 and 5. Social anxiety symptoms were also predicted by HR, lnHF, the α1 exponent, and entropy from scale factors 5 to 10. The α2 exponent predicted panic, obsessive-compulsive, and total anxiety symptoms. Obsessive-compulsive and total anxiety symptoms were predicted by entropy at scale factors from 10 to 20. Separation anxiety symptoms were predicted by entropy at scale factors 5 and 10. Finally, depression was predicted by entropy at scale factor 20. If we look at the direction of these relationships, we see that the lower HRV or CC is, the higher internalizing symptoms are.

Interestingly, the regression analysis for both adjusted and unadjusted data showed that sex did not predict the self-reported scores, except in the case of social anxiety. For unadjusted data we found interaction effects between sex and the α1 exponent in the prediction of depression symptoms, and between sex and entropy at scale factors 5 and 10 in the prediction of separation anxiety symptoms. However, when we analyzed the adjusted indices, the interaction between sex and cardiac measures was not significant in any regression model, meaning that the effect of the cardiac measure on the internalizing symptoms did not differ by sex. Thus, even though adolescent females are more “physiologically” vulnerable, they did not feel more depressed or anxious than their male counterparts, except in social conditions. The greater social anxiety experienced by females might be influenced by several factors, such as different levels of pubertal maturation or social demands (for instance, adolescents spend several hours at school, a context where high social demands are common).

We might be able to understand the lack of sex differences in self-reported anxiety and depression from a diathesis-stress model at the onset of anxiety and depressive disorders. According to Christiansen (2015) and Hyde et al. (2008), biological sex differences result in an inherent vulnerability toward anxiety and depression in females, and cultural influences may further increase sex differences in developing specific IDs through the stress component of the diathesis-stress model (for a review, see Belsky and Pluess, 2009). When HRV is below a certain value, the individual might be more prone to developing an ID, but that ID would not be the unavoidable consequence of the physiological condition. For instance, girls may or may not show higher anxiety scores than boys, but they could still be more physiologically vulnerable to developing an ID due to their lower HRV and CC. However, whether they will develop an ID also depends on other factors, such as the contextual conditions in which they live.

To sum up, our results provide some evidence supporting the role of HRV and CC as relevant factors for the early identification of physiological vulnerability of adolescent girls. From a clinical point of view, the fact that girls and boys do not significantly differ in self-reported levels of anxiety or depression may mask the girls' vulnerability, and consequently, it might hinder the development of strategies to protect girls against anxiety.

Although we consider reduced HRV and CC as vulnerability factors for IDs, we are aware that a more global, neurovisceral view (see Thayer and Sternberg, 2006) is necessary to build a theory on physiological vulnerability to IDs. This view includes brain structures (e.g., the amygdala) and allostatic systems (e.g., the hypothalamic-pituitary-adrenal axis) where sex differences exist (Lenroot and Giedd, 2010). However, it is far beyond the scope of this report to undertake such a neurovisceral review.

It is also important to note that studies on healthy adults have reported that females' HRV and CC are usually greater than they are in males (Ryan et al., 1994; Koenig and Thayer, 2016). Since HR regulation changes throughout the lifespan (O'Connor et al., 2008; Moodithaya and Avadhany, 2012), further longitudinal research should focus on when these patterns invert along the course of development and why. Perhaps the higher HRV and CC in some adult females come from an effort to balance out anxiety or depression. In fact, Thayer et al. (1998) reported that females suffering from depression showed more heart period variability compared to their counterparts without depression. More recently, Williams et al. (2015) found that the relationship between 5-min resting HF-HRV and negative psychological states can be positive in women. Although this result could point to an overcompensation of heart regulation as a way to overcome anxiety or depression, it is insufficient in women with internalizing symptoms or disorders.

Concerning the clinical implications of the results of the present study, adolescence could be the right time to use strategies aimed at preventing IDs. However, we cannot base these strategies solely on self-reported anxiety scores since healthy adolescent girls do not show higher scores than their counterparts at that age (except in social anxiety, in our study). What is different, depending on sex, is HR regulation, which is significantly less variable and complex in females, and might be making them more prone to developing IDs. From physiologically based interventions (e.g., HRV biofeedback, see Lehrer and Gevirtz, 2014) to psychological treatments (e.g., cognitive behavioral therapies, see Garanaki et al., 2009), a wide range of procedures are available to set up and implement those preventive strategies in adolescent girls who might be “physiologically” vulnerable to IDs. The results of the current study should be helpful in identifying such girls. The regression models suggest that the lower the HRV or CC, the higher the risk for IDs.

We are aware of the controversy around the need to adjust for HR when measuring HRV. In order to shed light on this issue, we encourage future research to analyze adjusted and unadjusted HRV measures.

The current study has various limitations that should be considered in future studies. First of all, we have not addressed whether sex differences in HR regulation would arise in stressful laboratory conditions. Studies on vagal reactivity are important to better understand the physiological components of complex heart regulation in highly stressful situations, but we wanted to examine how adolescent males and females regulate their HR in common everyday situations, regardless of how stressful they perceive these conditions to be. The results obtained in reactivity studies could be biased since everyone reacts to stress, but differences in HRV and CC in daily life can more reliably reflect “physiological” vulnerability.

Secondly, we did not monitor participants' activities while the cardiac devices were recording. We assumed that these activities (usually listening to teachers or working in the classroom) would be equivalent, though we cannot be sure. Lastly, we did not register the different phases of the menstrual cycle. Although we assessed menstruating participants on another day, it has been suggested that all menstrual stages have an effect on the brain areas related to HR regulation (Bäckström et al., 2011). However, in our opinion, the size of the group of females was large enough to ensure these limitations would not influence the results.

Despite these shortcomings, the present study uses a large sample of adolescents that is homogeneous with regards to the age range studied. Furthermore, the fact that adolescents were monitored under ecological conditions improves the external validity of the findings. Altogether, it makes the results of our study robust and helpful in comprehending how sex differences in HR regulation could contribute to the onset of internalizing disorders.

## Author contributions

AF-V has contributed in the study design, collection, analysis and interpretation of data, and in the writing of the manuscript. AD has contributed to the collection and analysis of data and in writing the manuscript. MB has contributed to the study design and editing the manuscript. XB is the main researcher of the more in-depth project where this study took place. He has contributed to the study design, analysis and interpretation of data, as well as in writing and editing the manuscript.

### Conflict of interest statement

The authors declare that the research was conducted in the absence of any commercial or financial relationships that could be construed as a potential conflict of interest.
